# Circadian Blood Pressure Rhythm in Cardiovascular and Renal Health and Disease

**DOI:** 10.3390/biom11060868

**Published:** 2021-06-11

**Authors:** Jiayang Zhang, Ruoyu Sun, Tingting Jiang, Guangrui Yang, Lihong Chen

**Affiliations:** 1 Advanced Institute for Medical Sciences, Dalian Medical University, Dalian 116044, China; zhangjiayang0912@163.com (J.Z.); sunryu07@163.com (R.S.); Jiangtdl@163.com (T.J.); 2 School of Bioengineering, Dalian University of Technology, Dalian 116024, China; guangrui@dlut.edu.cn

**Keywords:** circadian rhythm, blood pressure, cardiovascular, renal

## Abstract

Blood pressure (BP) follows a circadian rhythm, it increases on waking in the morning and decreases during sleeping at night. Disruption of the circadian BP rhythm has been reported to be associated with worsened cardiovascular and renal outcomes, however the underlying molecular mechanisms are still not clear. In this review, we briefly summarized the current understanding of the circadian BP regulation and provided therapeutic overview of the relationship between circadian BP rhythm and cardiovascular and renal health and disease.

## 1. Introduction of Circadian BP Rhythm

**Figure 1:**
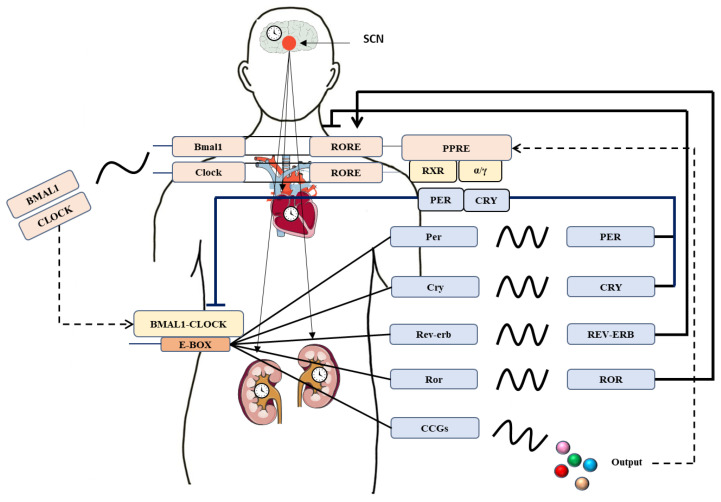
Molecular mechanism of circadian clock in humans. The molecular oscillator comprises interconnected transcription-translation feedback loops. The expression of CCGs is driven by the output of the molecular clock and adjusts circadian clock of cardiovascular and renal function. ROR activates and REV-ERB represses RORE-mediated transcription. PPARα and PPARγ regulate the expression of BMAL1 and REV-ERB via binding to PPRE in their promoters. As the PPAR partner, RXR inhibits the transcriptional activity of BMAL1-CLOCK complex. RORE, ROR response element. PPRE, PPAR response element. RXR, retinoid X receptor.

Circadian rhythm refers to an endogenous biorhythm with a 24 h cycle driven by an intrinsic and periodic molecular clock that adjusts behavior and physiological activities to external environmental changes [1,2]. In mammals, the master pacemaker located in the suprachiasmatic nucleus (SCN) of the hypothalamus elaborately controls the peripheral clocks in other tissues to ensure the synchronization of all existing clocks [3,4]. As is shown in Figure 1, the molecular circadian clock in mammals is driven by interactional transcription-translation auto-regulatory feedback loop with a subset of core clock genes including *Bmal1* (brain and muscle aryl-hydrocarbon receptor nuclear translocator-like 1), *Clock* (circadian locomotor output cycles kaput), *Per1/2/3* (period1/2/3) and *Cry1/2* (cryptochrome1/2). BMAL1 and CLOCK proteins collectively form a heterodimer to bind on E-box elements in promoter regions of clock-controlled genes (CCGs) including several important genes related to cardiovascular and renal function [5,6]. When the accumulation of PER and CRY proteins is fully superfluous in the cytoplasm, both of them translocate to the nucleus where shut down the transcription of heterodimeric activator [7]. In addition, nuclear receptors ROR (RAR-related orphan receptor) activates and REV-ERB (nuclear receptor subfamily 1, group D) suppresses RORE (ROR element) to directly control *Bmal1* and *Clock* transcription [8]. Moreover, another nuclear receptor PPARγ integrated in this loop is also a target gene of BMAL1 by directly binding to PPRE (PPAR response element) in its promoter [9]. Particularly, all of dynamic but coordinated PPAR isoforms displayed diurnal expression at different time point in mouse tissues [10]. It is estimated that almost 10–15% of the gene transcripts in the heart and kidney are under circadian transcriptional control [11].

Emerging evidence indicates that most cardiovascular and renal functional fluctuations are evolutionarily controlled by circadian clocks. BP, which is crucial to cardiovascular and renal health, displays obvious circadian rhythm with nocturnal dip and morning surge in both human and rodents [12]. The circadian pattern of BP can be affected by many factors.

For instance, melatonin, secreted from the pineal gland at night, may significantly reduce nocturnal BP and improve sleep in patients with essential hypertension [13]. Rhythmicity of atrial natriuretic peptide (ANP) appeared to be in antiphase with BP rhythm, which has been proven to serve as an important regulatory factor of the 24 h BP pattern and affect the cardiovascular system [14]. Circadian rhythms of plasma renin activity (PRA), angiotensin converting enzyme (ACE) activity, concentration of AngII, aldosterone and thyroid hormone contribute to the maintaining 24 h BP rhythm as well [15,16,17,18,19]. In addition to hormones, blood vessel components, such as smooth muscle cells and endothelial cells, and many vasoactive substances can change the diurnal variations of BP. Typically, nitric oxide (NO) secreted from endothelial cells modulates vascular tone and thus BP. NO production is increased in the morning, followed by a morning surge in BP. Yet, whether the diurnal variation of NO is directly related to BP rhythmicity is in debate. Disruption of the diurnal oscillation of NO production has been closely connected with BP alterations in cardiovascular disease [20]. Furthermore, endothelial NO synthase (eNOS) is one of the three NO synthase enzymes, which produce NO in blood vessels and regulate vascular function. In animal studies, the phosphorylated-eNOS (p-eNOS) in the blood vessels of young mice exhibited a circadian rhythm. The core clock genes can also regulate the coupling of eNOS and contribute to the maintenance of rhythmicity of endothelial function and BP [21]. Similarly, although 3.5-day rhythm and 8-hourly change have been reported in the human circulation [22], the secretion of endothelin 1 (ET-1) in plasma and excretion in urine has been also pronouncedly confirmed to be circadian rhythmic and closely related with BP homeostasis [23,24,25]. ET-1 receptor antagonists had been proved to be efficient for the treatment of essential hypertension, however, significant fluid retention and edema side effects were also reported [26]. Besides the above, there are many other endogenous factors contribute to the 24 h BP oscillations through humoral, endocrine, neural or other coordinated regulation signals. Furthermore, some diseases can alter the 24 h BP rhythm. For instance, patients with obstructive sleep apnea (OSA) are more likely to have non-dipper BP, which will increase the incidence of cardiovascular events [27].

For exogenous aspect, growing studies have revealed that the sleep patterns may have significant effects on BP day-night profile [28]. For instance, people with sleep disturbance or circadian misalignment by shift work and social jet lag often suffer from hypertension [29], BP phase delay [30], abnormal rhythm of melatonin secretion [31] or increased high-sensitivity C-reactive protein [32]. Furthermore, although our own studies have illustrated that the diurnal rhythms of BP are subject to more direct control by the clock than behavior, at least under basal conditions [33,34]. The most recent study, however, found that the timing of food intake can entrain the diurnal BP rhythm independent to *Bmal1* and the light-dark cycles [35]. In addition, the rhythm of BP can be affected by temperature, noise and many other environmental factors [36,37,38].

## 2. Canonical Clock Genes and BP Regulation

As mentioned above, the circadian rhythm of BP is driven by a complicated molecular clock network. Growing evidence from rodents has found that the circadian rhythms of BP can be distinctly altered by genetic manipulation of the core clock genes (Table 1).

### 2.1. Bmal1

Several studies indicated that mice of *Bmal1* deletion exhibited altered BP and (or) impaired cardiovascular and renal functions [21,39,48,51,52,54,55,56]. Loss of diurnal variation in BP accompanied by hypotension phenotype has been confirmed in both prenatal and postnatal *Bmal1* global knockout mice [33,39]. On the contrary, although *Bmal1* knockout rats resulted in an overall lowering of arterial pressure, the BP rhythms remained intact [40]. Differences between species may be a possible explanation. Specific depletion of *Bmal1* in mice from smooth muscle cells (SM22α-Cre), but not from cardiomyocytes, significantly suppressed the amplitude and shifted the acrophase of BP oscillation [48]. Mechanistically, BMAL1 regulates the time-of-day variations of Rho-kinase 2 (ROCK2) activity by directly binding on its promoter, and *Bmal1* deletion greatly diminished ROCK2 activation, myosin phosphorylation, and then the diurnal variations in response to vasoconstriction [48]. Chang and colleagues showed that loss of *Bmal1* in perivascular adipose tissue reduced BP in mice during the resting phase [49]. Moreover, mice with depression of *Bmal1* in endothelial cells (TEK-Cre) had significantly lowered mean arterial pressure as well, however the molecular mechanism is still not clear [50]. In terms of renal *Bmal1* in circadian BP regulation, Firsov and colleagues have confirmed a mild decrease in systolic BP, but not diastolic BP, with intact circadian rhythm in the mice lacking *Bmal1* in the entire nephron (Pax8-Cre) [51]. While in another mouse model where *Bmal1* was depleted in the renin-secreting cells (Ren1-Cre), reduction in the amplitude of BP was also observed, which is accompanied with many other renal abnormalities including the changes in the circadian rhythm of urinary sodium excretion and increased glomerular filtration rate, and reduced plasma aldosterone levels [52]. In addition, Pollock and colleagues have shown that loss of *Bmal1* in the collecting duct (AQP2-Cre) significantly lowers BP in male, but not female mice compared to control mice [53]. Gumz and colleagues showed mice lacking *Bmal1* in the thick ascending limb, distal convoluted tubule, and collecting duct cells (Ksp-Cre) displayed an obviously lower basal systolic BP compared to control mice [54]. Taken together, *Bmal1*, especially expression in cardiovascular and renal system, plays critical role in maintaining BP homeostasis and rhythm.

### 2.2. Per

Most recently, a series of studies from rodents have elegantly clarified the important role of *Per1* in BP regulation and electrolyte handling. Radiotelemetry recordings and Elisa measurement showed that mice lacking *Per1* exhibited significantly reduced BP and elevated renal ET-1 levels in male 129/sv mice [43]. However, altered ET-1 levels were not observed in female *Per1* knockout mice with the C57BL/6 background [57]. High salt diet plus mineralocorticoid deoxycorticosterone pivalate (HS/DOCP) injection significantly resulted in non-dipping hypertension and renal sodium handling defect (<10% difference between active and inactive phase) in male *Per1* knockout mice [44,58]. Interestingly, the female *Per1* knockout mice appeared to be protective against non-dipping hypertension in response to HS/DOCP treatment in sex-dependent manner to some extent [57]. Moreover, in contrast to C57BL/6 background, male *Per1* KO mice on a 129/sv background in combination with HS/DOCP challenge are extraordinarily protective from hypertension [59], implying that sexual hormones might be fundamental to *Per1* medicated circadian BP control. The complexity of *Per1* in circadian BP control may need further investigation. In accordance with altered BP pattern, the clock gene *Per1* might regulate the transcription of epithelial sodium channel (*ENaC*), sodium-glucose linked transporter-1 (*SGLT-1*), sodium-hydrogen exchanger-3 (*NHE3*), endothelin-1 (*ET-1*), which are responsible for sodium transport in the kidney [43,60,61]. As for another isoform of gene *Per*, *Per2* mutant mice showed a mild cardiovascular phenotype with an elevated 24 h heart rate as well as decreased 24 h diastolic BP during the active phase [46]. However, the systolic BP and mean arterial BP displayed no significant difference between the mutant and control mice [46]. In addition, *Per2* mutant mice resulted in mild attenuation of 24 h BP rhythm, heart rate, and locomotor activity with much shorter circadian periods than wild-type mice under constant dark condition [46]. Collectively, the clock component *Per* acts as a crucial mediator in circadian system to be involved in BP regulation.

### 2.3. Clock and Cry

It has been reported that the *Clock* mutant mice on the Jcl/ICR background displayed dampened diurnal rhythm of BP and heart rate [42]. While in *Clock* knockout mice, the arterial BP was normally rhythmic and significantly lower with about 10 mmHg decline compared to control mice, accompanied by mild diabetes insipidus, impaired sodium excretion rhythm [41]. With regard to another core clock component *Cry*, Doi et al. have unveiled that simultaneously lacking *Cry1* and *Cry2* in mice would lead to salt-sensitive hypertension due to abnormally chronic over-production of the mineralocorticoid aldosterone by the disordered adrenal gland [47]. DNA microarray further revealed that overexpression of a specific enzyme *Hsd3b6* may contribute to the high mineralocorticoid aldosterone synthesis in *Cry*-null mice, implying that clock-dependent sodium excretion may play an important role in the BP circadian homeostasis [47]. However, the causal connection between altered sodium excretion and BP regulation needs to be further explored.

It is worth mentioning that in addition to the core clock gene manipulation models, the transgenic hypertensive TGR(mREN2)27 rat, which harbors an additional mouse renin gene and exhibits an inverse circadian BP profile, has also been commonly used for circadian BP rhythm studies [62,63,64].

## 3. Disruption of Circadian BP Rhythm and Cardiovascular Disease

BP rhythm is one of the most common circadian rhythms in the cardiovascular system [65]. Disruption of the BP rhythm has been considered to be a major contribution to many adverse cardiovascular events. It has been found that every 5% drop in the decline in nocturnal BP may increase the risk of cardiovascular death by about 20% [66].

Myocardial infarction (MI) is an acute heart disease, which can be complicated by arrhythmia, shock or heart failure, and is life-threatening. There is an inseparable connection between the rhythm of BP and MI. Abnormal BP circadian patterns or chronic hypertension may lead to changes in cardiac hemodynamics, and increase the incidence of MI [67]. Several Clinical studies have proved that the incidence of MI is rhythmic and significantly increased incidence rate was usually observed between 06:00 am and 12:00 pm which is somehow consistent to the BP morning surge. As for systolic BP, lower (<100 mm Hg) in post-MI period increase the risk of cardiovascular events, while patients with elevated systolic BP (>140 mm Hg) have a higher risk of stroke and combined cardiovascular events [68]. Compared with resting systolic BP, systolic BP after exercise can provide higher predictive value for acute MI [69]. In terms of diastolic BP, acute myocardial infarction (AMI) patients with lower diastolic BP (<70 mmHg) have increased postoperative risks, including all-cause death, cardiovascular death and cardiovascular hospitalization. Moreover, wide pulse pressure was also considered as a predictor of MI [70]. Furthermore, several core clock genes are considered as important components in regulating the rhythmic pathogenesis of MI. There is an association between MI and *Clock* and *Arntl* (also known as *Bmal1*) gene polymorphisms [71], and the genetic variants of *Arntl*, *Clock* and *Per2* genes are proved to be related to circadian phenotype (i.e., chronotype or daytime sleepiness) in patients with MI [72].

Under the condition of heart failure with preserved ejection fraction, the increased variability of systolic and diastolic BP is related to the increase of adverse events [73]. Concurrently, abnormal systolic BP and diastolic BP can cause the variation of heart indicators. As the systolic BP increases, the left ventricular wall thickness and left ventricular mass index also increase with the higher diastolic BP; while the functional index of left ventricular diastolic function is inversely proportional to the increase of diastolic BP, but irrelevant with systolic BP [74]. Overall, there is a U-shaped relationship between systolic BP and heart failure, that is, lower or higher systolic BP will lead to higher risk of death and heart failure hospitalization [75]. The same phenomenon exists in diastolic BP [76]. Therefore, when diagnosing MI and related cardiovascular events, it is necessary to comprehensively consider the phenotype and rhythms of BP.

Given the significant correlation between acute cardiovascular events and BP rhythms, it is necessary to improve the therapeutic strategies according to the characteristics of circadian rhythm changes. In addition to optimizing the drug administration time, it has been reported that the time of cardiovascular surgery may have influences on the tissue repair and outcome of cardiovascular diseases as well. For example, although the most recent studies demonstrated that the time-of-day variation of aortic valve replacement surgery had no significant impact on clinical outcomes and had no clinically relevant biorhythm for myocardial ischemia-reperfusion tolerance [77,78,79]. Montaigne et al. did have reported that the incidence of major adverse cardiac events in the afternoon surgery group was significantly lower than that in the morning group [80]. More interestingly, in patients undergoing non-cardiac surgery, Lavallaz et al. found that the incidence of AMI during follow-up is instead increased in the afternoon surgeries [81]. Nevertheless, a better understanding of the relationship between circadian clock and cardiovascular disease may help to develop more targeted and personalized treatment strategies for patients.

## 4. Disruption of Circadian BP Rhythm and Renal Damage

Chronic kidney disease has long been closely linked with sleep disturbance and hypertension and thus gradually become a worldwide health problem with a great economic burden [82,83]. In humans, accumulating clinical evidence indicated that blunted BP variation from 24 h BP recordings was associated with increased risk of end-organ damage and progressive loss of renal function [84,85]. Predictably, the consistency of circadian BP variation was ruined in chronic kidney disease stages 3–5 [86]. In addition, among patients with chronic kidney disease, circadian BP profile is closely related to the level of physical activity and the severity of target organ damage [87]. Moreover, clinical trials indicated that circadian BP profile is associated with mortality among elderly veterans with chronic kidney disease [88]. Meanwhile, the nocturnal dip in BP is nearly diminished in patients with glomerulopathy, resulting in enhanced urinary excretion rate of sodium and protein during the inactive phase [89].

To date, an explicit mechanism has not been expatiated between chronic kidney disease and disruption of circadian BP rhythm. In rodent models, studies have found that after treatment with adenine for 2 to 4 weeks to induce chronic kidney disease in mice or Sprague–Dawley rats, the animals will display significantly increased arterial BP and disrupted day-night variation [90]. In addition, the SCN of PER2::LUC mice with adenine-induced chronic kidney disease displayed dampened amplitude rhythms in their central circadian clock as measured by bioluminescence. Meanwhile, the kidney explants from chronic kidney disease mice expressed altered PER2::LUC rhythms with significantly longer period compared to control mice [91]. On the other hand, upon adenine treatment, *Clock* mutant mice showed significantly higher BP and worse renal function than control mice, implying the circadian BP disruption enhances the susceptibility to chronic kidney disease [91]. However, our own recent study found that postnatal *Bmal1* deletion in mice protected against renal fibrosis via suppressing *Gli2* transcription, which provides a prominent role of the core clock gene *Bmal1* in tubulointerstitial fibrosis [92]. Nevertheless, more efforts need to be made from different point of view to further clarify the relationship between circadian BP disruption and chronic kidney diseases.

## 5. Chronotherapy of Anti-Hypertensive Medications

Altered day-night oscillation in BP has been associated with high morbidity and mortality of cardiovascular disease and increased progression of kidney damage. Of note, non-dipping hypertension is frequently observed in clinical outcomes of chronic kidney disease and associated with increased risk of cardiovascular event [93]. While an excessive circadian variation in BP is also associated with an increased risk of nephropathy [94,95]. Thus, the application of anti-hypertensive drugs, including angiotensin-converting enzyme inhibitors (ACEI), angiotensin II receptor blockers (ARB) and others, becomes fundamental to the prevention and treatment of many cardiovascular and renal diseases. The rational and effective administration time of these anti-hypertension medications has also been explored and reported in many clinical trials and animal studies.

### 5.1. ACEI

Clinical studies demonstrated that bedtime administration of the long-acting lipophilic ACEI trandolapril was a safer and more effective mean of morning BP control in hypertensive patients compared to morning-dosed group [96]. The combination medication of captopril and hydrochlorothiazide was mildly more effective in nighttime BP reduction when ingested in the evening in 13 male patients with moderate hypertension for 3 weeks [97]. In addition, bedtime spirapril administration was more efficient than morning administration in reducing asleep BP, which might be associated with the nocturnal RAAS activation [98]. Additionally, evening treatment of enalapril would further decrease nighttime BP followed by a slow increase during the day in a randomized crossover design [99]. Furthermore, lisinopril administration at 10.00 pm. has been shown to be much more useful [100]. With regard to quinapril, the evening administration resulted in similarly more preferable antihypertensive effect, whereas the morning schedule represented a smaller reduction in nighttime BP [101]. However, there are also examples of the opposite, in a single-blinded crossover study of 10 hypertensive subjects, which have been reported that morning administration of benazepril had a more sustained anti-hypertensive effect than evening administration [102]. Moreover, although the hemodynamics seemed better after the evening intake of the ramipril medication, the hypotensive effect of ramipril and imidapril appeared approximately the similar between morning administration and evening administration in a crossover study involving patients with hypertension [103,104].

### 5.2. ARB

ARBs are becoming more popular for the treatment of hypertension and highly effective and well tolerated. Valsartan administration at bedtime, as opposed to upon awakening, resulted in a highly significant average increase by 6% in the day-night BP difference toward a more dipper pattern, which corresponded to a 73% relative reduction in the number of patients with non-dipping hypertension [105,106]. Furthermore, it has been found amlodipine and valsartan combination therapy should be preferably administrated at bed time for the majority of controlled patients with potential additional cardiovascular event occurrence reduction [107]. However, in subjects with requirement to combined medication to achieve proper BP-lowering effect, co-administration of amlodipine and valsartan efficiently reduced BP for the entire 24 h independent of dosing time compared to any one of the other assessed therapies [107]. Studies also demonstrated that telmisartan administered at bedtime improved the sleep time-relative BP decline and nocturnal BP regulation compared to dosing upon awakening [108]. Olmesartan, another angiotensin II type 1 receptor blocker, was also shown to retain night-time BP dip possibly by enhancing day-time sodium excretion to relieve cardio-renal load through normalization of circadian BP rhythm [109,110,111]. However, although evidence has shown administration of olmesartan at bedtime was significantly more efficient in reducing the nocturnal BP than morning administration [112], controversial observations have also been reported in some trials [113].

### 5.3. Other Anti-Hypertension Medications

Several prospective trials have been reported to elucidate the protective effect on low-dose aspirin in patients suffering from hypertension only administrated at bedtime, beyond the secondary prevention of cardiovascular disease [114,115,116,117,118]. Likewise, we have recapitulated the paralleled time-dependent hypotensive effect on hypertensive mice treated with low-dose aspirin [119], which could provide a beneficial approach to solve the BP control of hypertensive subjects throughout the day. Additionally, nifedipine is also marked as an anti-hypertensive agent. The BP reduction after nifedipine treatment was significantly larger with bedtime administration than morning ingestion, as well as efficiently reduced the incidence of edema and the total number of adverse effects compared to ingestion of nifedipine on awakening [120,121,122].

Taken together, although growing evidence demonstrated that bedtime administration of the current anti-hypertension medications would be more efficiently in reducing BP, however there are also many inconsistencies. Indeed, in the most recent Hygia Chronotherapy Trial, which examined the effects of bedtime vs. upon-waking BP-lowering treatment on cardiovascular risk in a large cohort with 19,804 participating patients, although authors claimed that routine bedtime ingestion of more than one BP-lowering medications may result in improved BP control and diminished occurrence of adverse cardiovascular events [123], this study only included patients from one population, the reduction of BP was relatively small, and it seemed no properly randomized controlled trial were performed in this big trial [124]. In addition, in a trial with patients of hypertension-related chronic kidney diseases, Rahman et al. also failed to observe any difference between the bedtime and morning antihypertensive treatment on BP control [125]. These inconsistencies may reflect the differences in cohort or drug selection or the variations in BP monitoring methodologies. However, attention may have to be brought to the limitations of recommending a single optimal time for an entire population, without concern for the chronodiagnosis and effects of the anti-hypertensive medications on the circadian amplitude and phase of BP. Some, but not all anti-hypertensive medications affect the circadian amplitude of BP [126]. Nevertheless, further in-depth exploration is required for the chronotherapy of anti-hypertensive medications. Merits of personalized chronotherapy that account for the chronodiagnosis have been documented [127].

## 6. Conclusions and Perspectives

Growing evidence indicates that the maintenance of BP circadian rhythm is highly associated with the cardiovascular and renal homeostasis, many anti-hypertension medications displayed clear circadian time-dependent efficiency and chronotherapeutic evaluation to the management of hypertension and the related cardiovascular and renal diseases had be taken into consideration for many studies. However, further research in this field still has to lay emphasis on accurate pharmacodynamics and pharmacokinetics of time-dependent administration by using novel compounds that modulate circadian timing system to superiorly prevent the onset and progression of hypertension and the related cardiovascular and chronic kidney diseases, which will provide a valuable and cost-benefit approach for BP control and potential merit of chronotherapy, as well as added systematic protection.

Moreover, the molecular link between the circadian BP regulation and cardiovascular and renal functions is still less known. RNA-seq analysis revealed that heart and kidney are two of the top five candidates along with the liver, lung and brown fat following obvious circadian regulation. However, the complexity of the cellular components and molecular network in the heart and kidney largely renders the mechanism exploration. Further study by tissue or cell specific depletion of the circadian genes in the heart and kidney may provide a better understanding of the relationship between BP rhythm and cardiovascular and renal function and pave a way for preventing the development of cardiovascular and renal diseases.

## Figures and Tables

**Table 1:** The effect of the core clock gene manipulation on BP phenotypes in rodents.

**Gene Editing**	**Species**	**Position**	**Phenotype of BP**	**Circadian Rhythm** **of BP**	**Reference**
*Bmal1* KO	mouse	global	↓	No	[33,39]
*Bmal1* KO	rat	global	↓	Yes	[40]
*Clock* KO	mouse	global	↓	Yes	[41]
*Clock* mutant	mouse	global	Day-night variation↓	Yes	[42]
*Per1* KO (129/sv)	mouse	global	↓	Yes	[43]
*Per1* KO (C57BL/6)	mouse	global	↑	Yes	[44]
*Per2* KO	mouse	global	=	Yes	[45]
*Per2* mutant	mouse	global	DBP↓	Yes	[46]
*Cry1/2* KO	mouse	global	Salt-sensitive hypertension	No	[47]
*Bmal1* KO	mouse	smooth muscle	↓	Yes	[48]
*Bmal1* KO	mouse	brown adipocytes	↓	No	[49]
*Bmal1* KO	mouse	endothelial cells	↓	Yes	[50]
*Bmal1* KO	mouse	tubular cells	SBP↓	Yes	[51]
*Bmal1* KO	mouse	renin-secreting cells	↓	Yes	[52]
*Bmal1* KO	mouse	collecting duct	↓	Yes	[53]
*Bmal1* KO	mouse	thick ascending limb, distal convoluted tubule, and collecting duct	↓	Yes	[54]

Phenotype and circadian rhythm of BP compared to control mice under 12:12 L:D cycles. ↓ means decline; ↑ means increase; = means no difference; SBP, systolic BP; DBP, diastolic BP.

## Data Availability

Not applicable.
